# Indirect bioleaching for metal recovery from battery black mass using microbially generated acid from a secondary sulfur resource

**DOI:** 10.3389/fmicb.2026.1921494

**Published:** 2026-08-27

**Authors:** Lalropuia Lalropuia, Karri Meriläinen, Wadih Y. Rassy, Eva Pakostova, Georg M. Guebitz

**Affiliations:** 1 K1-MET GmbH, Linz, Austria; 2 Department of Agricultural Sciences, Institute of Environmental Biotechnology, BOKU University, Tulln an der Donau, Austria; 3 Biotechnology and Chemical Engineering, Turku University of Applied Sciences, Turku, Finland; 4 Department of Science and Technology, Institute of Biotechnology, IMC University of Applied Sciences, Krems, Austria; 5 Faculty of Technical Chemistry, TU Wien, Vienna, Austria; 6 School of Natural Sciences, Laurentian University, Sudbury, ON, Canada

**Keywords:** biogenic acid, bioleaching, biosurfactants, critical metals, hydrogen peroxide, lithium-ion batteries, recycling

## Abstract

Recycling critical metals from spent lithium-ion batteries (LIB) has attracted significant attention in recent years, driving the search for more sustainable and environmentally friendly recovery methods. Among emerging approaches, indirect bioleaching may be a very promising alternative for recovering valuable metals from spent LIB. In this study, sulfuric acid (H_2_SO_4_) was produced by using a mixed culture containing *Acidithiobacillus thiooxidans* and *Acidithiobacillus caldus*, supplemented with elemental sulfur recovered as a waste product from a biogas plant. The biogenic acid was subsequently used for leaching black mass derived from nickel manganese cobalt (NMC)-based lithium-ion batteries (LIB). First, the influence of different concentrations (0.2, 1, and 2 M) of hydrogen peroxide (H_2_O_2_) on metal dissolution was investigated. Subsequently, 2% (v/v) of rhamnolipids / phospholipids was added and the effect on the H_2_SO_4_–H_2_O_2_ system was studied. Finally, the best performing system was scaled up to a 1L reactor, achieving complete recovery of Mn and Li from 10% (w/v) NMC within 1 h. This result highlights the high potential of microbially produced H_2_SO_4_ as an effective leaching agent for recovering critical metals from spent LIB, while simultaneously valorizing a secondary sulfur waste stream, aligning with the broader objectives of circular and sustainable resource recovery approaches.

## 1 Introduction

The increasing demand for lithium-ion batteries (LIB) has been driven by their widespread use in electric vehicles (EVs), renewable energy storage systems, and consumer electronics. For instance, the number of EVs is expected to increase to 965 million in 2050 (Llamas-Orozco et al., 2023). Spent LIB pose significant environmental risks due to their toxic and flammable components, which can contaminate the environment if not properly managed. At the same time, LIB present a valuable secondary resource of critical and strategic raw materials such as Li, Co, Ni, and Mn. Therefore, efficient recycling of LIB could be essential to close material loops, mitigate environmental risks, and reduce primary mining of these materials (Zanoletti et al., 2024).

LIB recycling is currently performed mainly using pyrometallurgy and hydrometallurgy methods. Although these methods have high recovery efficiencies, they are associated with significant drawbacks including high energy inputs, use of harsh chemicals such as concentrated acids, and generation of secondary waste streams (Liu et al., 2024). In this context, bioleaching presents a promising alternative. Bioleaching is a biotechnological process that uses microorganisms or their metabolites to solubilize metals. Conventional bioleaching, which has been extensively applied to process low-grade sulphidic ores and gold concentrates, uses a direct approach that partially relies on the solubilization of valuable metals through direct microbial interaction with the solid substrates, often assisted by biofilm and exopolysaccharide layer (EPS) formation (Vera et al., 2013). However, the application of direct bioleaching to extract metals from complex materials such as LIB often faces inhibitions of microbial growth and metabolic activity by toxic metals, electrolytes, binders, and other components present in the waste stream. Furthermore, direct bioleaching is constrained by challenges associated with high pulp densities and often require external pH adjustments to maintain conditions favorable for microbial growth and activity (Kiskira et al., 2026). To overcome this limitation, indirect leaching is applied to separate the microbial generation of the leaching agents (i.e., organic/inorganic acids and/or Fe^3+^) from the subsequent recovery of valuable materials from LIB (Boxall et al., 2018; Pakostova et al., 2024). Increasing research attention has been directed toward the bioleaching of LIB with a range of innovation approaches being developed to improve metal recovery. Naseri and Mousavi (2024) reported that a step-wise addition of biogenic acid produced by *Acidithiobacillus (A.) thiooxidans* during bioleaching of spent LIB increased the leaching efficiency of Mn and Li by 50% and 38%, respectively, and reduced the leaching duration from 16 to 8 days, when compared with direct bioleaching. Kim et al. (2024) reported that an application of static magnetic field enhanced the leaching efficiency of Li during bioleaching of spent LIB using *A. ferrooxidans*, a magnetotactic bacterium. High dissolution of Li (1.6 g/L) was achieved during bioleaching of LFP even at a high pulp density of 125 g/L and 89% of Li was recovered as Li_3_PO_4_ from the leachate solution by precipitation (Gu et al., 2025). The same study also determined that the bioleaching process has a lower carbon footprint, generating an average of 0.98 kg/CO_2eq_, compared with other battery recycling methods.

Sulfuric acid-mediated leaching is widely regarded as an effective approach for metal recovery from LIB black mass, offering high extraction efficiencies under relatively mild operating conditions (Lee et al., 2025). Biogenic sulfuric acid produced via microbial oxidation of reduced sulfur compounds represents a promising alternative to industrially produced sulfuric acid, as using biogenic acids reduces the carbon footprint and environmental hazards associated with chemical acid production. Successful applications have been reported by our group and others for valorization of metals from different waste streams such as waste incineration residues and pharmaceutical blisters (Kremser et al., 2022, 2023). Indirect leaching of LIB using biogenic sulfuric acid has been reported (Boxall et al., 2018; Pakostova et al., 2024), but it remains underexplored, particularly in relation to the role of chemical and biological additives in improving metal dissolution kinetics and process efficiency.

LIB encompasses a variety of cathode chemistries such as lithium nickel manganese cobalt oxide (NMC), lithium iron phosphate (LFP), and lithium cobalt oxide, with each type optimized for specific performance requirements (Manthiram, 2020). Although Co-free cathode materials such as LFP have recently been introduced, NMC has historically dominated the battery market predominantly used in EVs due to their high energy density (Fallah and Fitzpatrick, 2023). This means that NMC will constitute the largest fraction of LIB waste in the near future. In addition, NMC cathodes contain significant quantities of critical metals such as Li, Ni, Co, and Mn, making them attractive targets for resource recovery.

In NMC-based cathode materials, transition metals are present as mixed metal oxides (LiNi_*x*_Mn_*y*_Co_*z*_O_2_). Efficient dissolution of NMC requires acidic conditions, ideally in combination with a reducing agent or catalytic pathway, owing to the inherently high oxidation states and structural stability of the metal oxides (Meshram et al., 2016). In NMC, the transition metals are commonly present in their thermodynamically stable higher oxidation forms, with Mn mainly as Mn^4 +^, Co as Co^3 +^, and Ni as a mixture of Ni^2 +^/Ni^3 +^ (and Ni^4 +^ in charged states). These species exhibit limited solubility under acid-driven leaching alone. Reducing agents such as hydrogen peroxide (H_2_O_2_) facilitate the reduction of Mn^4 +^ and Co^3 +^ to the more soluble Mn^2 +^ and Co^2 +^ (while also assisting in the reduction of higher-valent Ni species), accelerating thus overall metal dissolution kinetics. In acidic media, H_2_O_2_ is oxidized to molecular oxygen, acting as a reducing agent for metal ions such as Co^3+^ (Lee and Rhee, 2003). As a result, introducing a reducing agent or catalytic species is required to facilitate redox reactions that convert these metals into more soluble oxidation states, thereby improving overall leaching kinetics. Importantly, the concentration of H_2_O_2_ is critical for efficient metal leaching; insufficient H_2_O_2_ limits the reduction of metal ions in higher oxidation states, whereas excess H_2_O_2_ may decompose to water and oxygen, reducing redox efficiency and potentially altering the solution potential. Optimization of H_2_O_2_ dosing is thus essential to maximize metal extraction. Equation 1 schematically represents the acid- and peroxide-assisted leaching of NMC material (Vieceli et al., 2023).


$${\text{LiNi}}_{\text{x}}\,{\text{Mn}}_{\text{y}}\,{\text{Co}}_{\text{z}}\,{\text{O}}_{2}+{\text{H}}_{2}\,{\text{SO}}_{4}+{\text{H}}_{2}\,{\text{O}}_{2}\,\to {\mathrm{NiSO}}_{4}+{\text{MnSO}}_{4}+$$ (1)

$${\text{CoSO}}_{4}+{\text{Li}}_{2}\,{\text{SO}}_{4}+{\text{H}}_{2}\,\text{O}+{\text{O}}_{2}$$

In addition to reducing agents, surfactants can influence leaching kinetics and interfacial reactions by modifying surface chemistry and enhancing mass transfer. Commercial surfactants are predominantly petroleum derived and therefore present potential environmental risks. On the other hand, biosurfactants are produced by microorganisms and are biodegradable, making them more environmentally friendly and less harmful (Mishra et al., 2021). Biosurfactants such as rhamnolipids and phospholipids possess amphiphilic structures that enable adsorption onto solid surfaces, potentially increasing wettability and promoting particle dispersion. These effects can enhance contact between the acidic leach liquor and the substrate surface, thereby facilitating metal dissolution and associated redox reactions at the solid–liquid interface (Roy, 2018). Additionally, biosurfactants may form weak complexes with dissolved metal ions, influencing metal speciation and transport in solution (Chernyshova et al., 2023). Biosurfactants have been used for different applications such as removal of metals from contaminated sites (Mulligan et al., 2001; Diaz et al., 2015; Mishra et al., 2021). Despite their demonstrated effectiveness in bioleaching and metal remediation systems, the role of biosurfactants in LIB black mass leaching has not yet been systematically examined.

In this study, microbially generated sulfuric acid produced from waste sulfur was evaluated for leaching valuable metals from NMC-based black mass. The effect of varying H_2_O_2_ concentrations on the extraction efficiencies of Ni, Mn, Co, and Li was systematically investigated to determine the optimal reducing agent dosage. Subsequently, two biosurfactants, rhamnolipids and phospholipids, were introduced into the bio-generated H_2_SO_4_ system under the optimized H_2_O_2_ conditions to assess their influence on metal dissolution. The best-performing system was then applied in a scale-up (1 L) leaching experiment at high pulp density and room temperature. To the best of the authors’ knowledge, the combined use of biogenic acid derived from waste sulfur with a systematic evaluation of H_2_O_2_ and biosurfactants for the leaching of black mass under ambient conditions and without external heating has not previously been reported. This integrated approach demonstrates a low-energy route for LIB recycling and contributes to the development of more sustainable metal recovery processes.

## 2 Materials and methods

### 2.1 NMC-based active cathode material

Black mass (BM) derived from lithium nickel manganese Cobalt Oxide (NMC) cathode material was obtained from a commercial recycling company in Germany. Metals contents in the BM were determined by inductively coupled plasma mass spectrometry (ICP-MS), in accordance with ÖNORM EN 13657:2002-12 (Table 1). In addition to the elements listed in Table 1, the NMC based BM consisted of C, which constitute a majority of the BM and traces of other elements.

**TABLE 1:** Elemental composition of NMC-based black mass.

Elemental concentration (wt %)						
Ni	Mn	Co	Li	Cu	Al	Fe
5.86	4.17	14.50	2.76	3.50	5.26	0.57

### 2.2 Biogenic H_2_SO_4_ production

A mixed culture of *A. thiooxidans* DSM 1927 and *A. caldus* DSM 9466 (1:1) both purchased from the German Collection of Microorganisms and Cell Cultures GmbH (DSMZ) were first cultivated separately, each in a 250 mL Erlenmeyer flask for 21 days at 30 °C and 150 rpm in an Infors HT Multitron Pro shaker (Bartelt, Austria). For the biogenic acid production, the two grown cultures were mixed (1:1) (v/v) and inoculated into a 2 L reactor (Schott DURAN, Germany) with a working volume of 1.8 L using basalt salt medium (pH 3.5) supplemented with 1% (w/v) sulfur. The basalt medium was prepared as described by Ňancucheo et al. (2016). The sulfur in a form of a dried powdered product was obtained from the desulfurization unit of a biogas plant in Denmark. The reactor was maintained at 30 °C and agitated at 250 rpm using a magnetic stirring plate (IKA RCT Digital, Germany) connected to a pH monitor and 50 L/h supply of pressurized air. After 14–21 days of cultivation, 1.2 L of the solution was removed and replaced with a fresh medium and S, with the remaining 0.6 L serving as an inoculum for the new batch. The collected solution was then centrifuged at 9,000 rpm for 20 min (SORVALL LYNX 6000, Thermo Scientific, Germany) to remove the solid residue and the biogenic acid was then stored at 4 °C. The acid production was carried out over a total of 10 cycles, yielding approximately 12 L of biogenic H_2_SO_4_ solution. The accumulated acid was subsequently used directly, without any dilution, adjustment, or other modification, in all leaching experiments reported in this paper. The acid concentration was determined by ion chromatography using Dionex ICS-900 (Thermo Fisher Scientific, United States) and confirmed by acid-base titration using phenolphthalein as indicator (Fluka Analytical) and 0.1 M NaOH as the titrant.

### 2.3 Indirect bioleaching

Batch indirect bioleaching was performed in 100 mL Erlenmeyer flasks (50 mL working volume) containing biogenic acid and 10% (w/v) NMC, placed on a magnetic stirring plate (VARIOMAG Poly, Thermo Scientific, Germany). All experiments were carried out in duplicate for 2 h at room temperature (22 °C) and 250 rpm, with samples being withdrawn every 20 min for further analyses. Different concentrations of H_2_O_2_ (0.2/1/2 M) were tested to investigate the effects of the reducing agent on the metal leaching efficiency. Further, the effects of two biosurfactants, namely 2% (v/v) rhamnolipids (RL) (AGAE Technologies, USA) and 2% (v/v) phospholipids (PL) (Soy Phospholipids, Sigma-Aldrich), were tested in combination with the optimal concentration of H_2_O_2_. For this, 10% NMC was added to the H_2_SO_4_-H_2_O_2_ solution and the biosurfactants (PL/RL) were added after 40 min, after which the experiment continued for 2 h.

Finally, the leaching system exhibiting the best performance was scaled up to a 1 L reactor comprising a jacketed reaction vessel with a flat bottom (DN 100, Schmizo, Switzerland), equipped with 2- blade impeller connected to a mechanical stirrer (Heidolph RZR 2021, Germany) and a condenser to prevent liquid loss due to evaporation. Sampling was performed at regular intervals in the first 2 h, and the experiment further continued for 24 h. The scaled leaching experiment was carried out in duplicate at room temperature and 250 rpm.

### 2.4 Analytical methods

Oxidation reduction potential (ORP) and pH measurements were regularly conducted using Inlab Redox (vs. Ag/AgCl) and LE422 electrodes respectively, both connected to a S220 pH/ion meter (Mettler Toledo, Switzerland). Liquid samples collected during leaching experiments were filtered using 0.2 μm polyamide syringe filters (Macherey-Nagel, Germany) prior to metal concentration determination by inductively coupled plasma – optical emission spectrometry (ICP-OES), using a 5110 ICP-OES connected to a SPS4 Autosampler (both Agilent Technologies, United States). The leaching efficiency of metals were calculated using Equation 2. The post-leaching solid residues were analyzed using a TM 3030 scanning electron microscope (SEM) equipped with an energy-dispersive X-ray spectroscopy (EDS) detector (Hitachi, Japan).


$$Leachingefficiency\left(\%\right)=\left[\left(C_{L}-C_{B}\right)/C_{N\,M\,C}\right]\times 100$$ (2)

where, C is metal concentration in leachate solution (C_*L*_), biogenic acid (C_*B*_) and NMC black mass (C_*NMC*_)

## 3 Results and discussion

### 3.1 Biogenic H_2_SO_4_

Biogenic sulfuric acid produced by a co-culture of *A. thiooxidans* and *A. caldus* cultivated in the presence of biogas plant derived sulfur reached a concentration of 0.63 M and a pH of 0.44 and was subsequently used in the indirect leaching experiments. Kremser et al. (2023) reported that the elemental sulfur derived from a biogas plant exhibited faster oxidation by a mixed culture of *A. thiooxidans* and *A. caldus* compared with commercial elemental sulfur. The same study also demonstrated improved Al leaching performance using the biogenic acid system. The authors attributed this enhanced performance to the presence of bioactive compounds in the bio-sulfur (Kremser et al., 2023). In a study by Välimets et al. (2026), biogas derived elemental sulfur was found to be more effective substrate than commercial sulfur for microbial sulfuric acid production. This was ascribed to its higher water solubility and the presence of organic compounds, such as carbohydrates and proteins, in the biogas-derived sulfur. Based on these findings, biogas-derived elemental sulfur was selected for the biogenic acid production, which was subsequently used in the leaching experiments conducted in this study.

### 3.2 Indirect leaching of NMC

**FIGURE 1:**
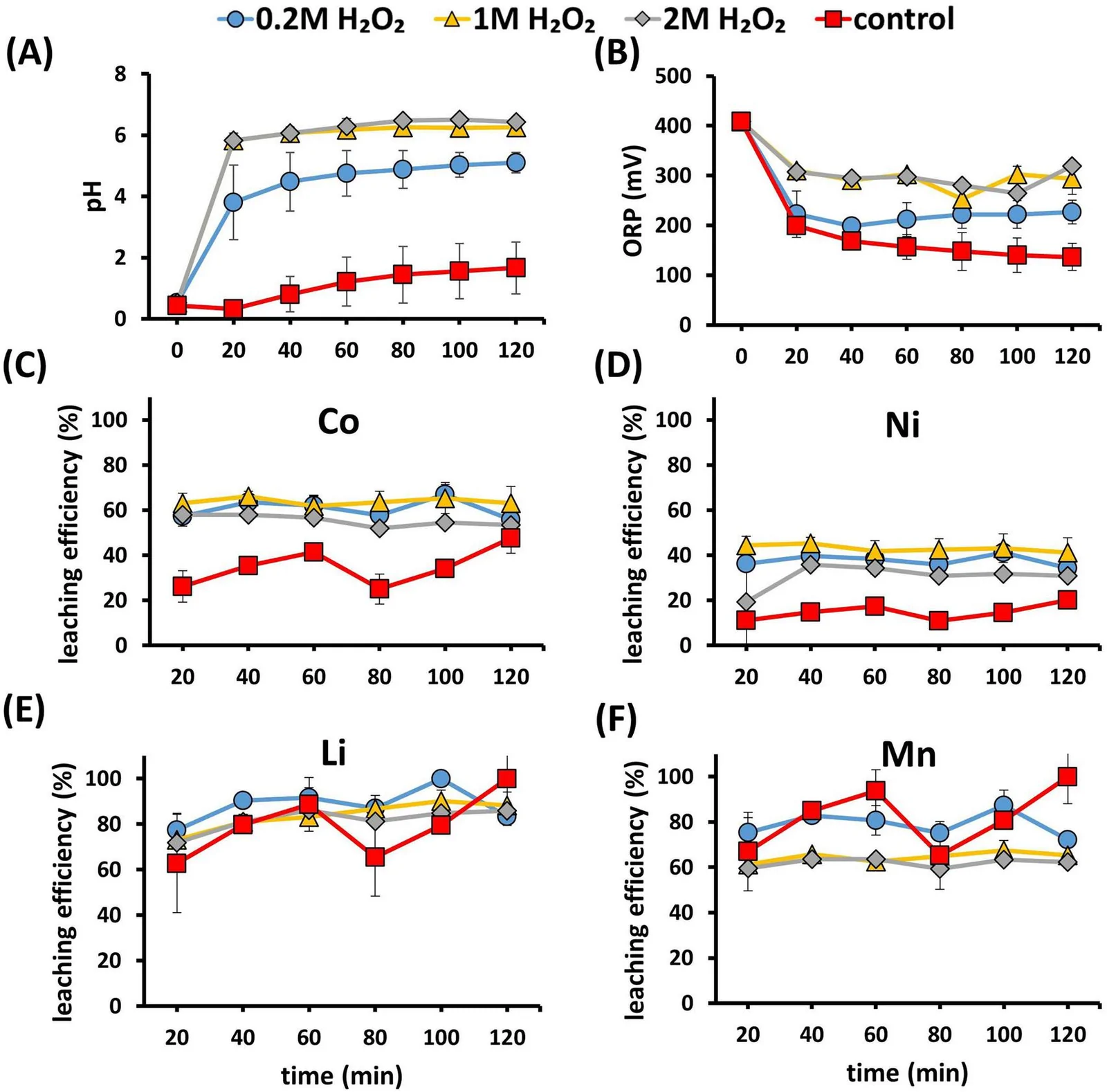
Changes in (A) pH, and (B) ORP, and leaching efficiencies of (C) Co, (D) Ni, (E) Li, and (F) Mn during indirect leaching of 10% (w/v) NMC using biogenic H_2_SO_4_ acid with different concentrations of H_2_O_2_ (0.2, 1, and 2 M). The error bars represent standard deviations (*n* = 2).

During indirect leaching of 10% NMC using biogenic H_2_SO_4_ supplemented with varying concentrations of H_2_O_2_, the addition of H_2_O_2_ generated strongly reducing conditions, as evidenced by the decrease in ORP. These conditions promoted the dissolution of lattice-bound Co^3+^ and higher-valent Ni species. At the same time, a gradual increase in pH was observed in H_2_O_2_-containing flasks (Figures 1A,B), reflecting proton consumption during reductive dissolution reactions and partial H_2_O_2_ decomposition. These changes were associated with improved extraction of Co (Figure 1C) and Ni (Figure 1D) relative to the H_2_O_2_-free control, confirming that acid dissolution alone is insufficient to efficiently disrupt the layered oxide structure of NMC materials. Of note, for Ni, moderate but consistent increase in recovery was seen upon H_2_O_2_ addition, which is consistent with the presence of reducible higher-valent Ni species within the cathode structure. In contrast, Li recovery remained consistently high (>80%) across all treatments, including the control, indicating that its dissolution is primarily governed by proton attack rather than redox processes, as previously reported (Xin et al., 2009).

Notably, increasing the H_2_O_2_ concentration above 0.2 M did not result in proportional improvements in metal recovery. This suggests that excess H_2_O_2_ undergoes rapid decomposition, reducing redox efficiency and potentially promoting secondary effects such as pH-dependent precipitation of dissolved species. H_2_O_2_ exhibits both oxidizing and reducing behavior, depending on the electrochemical environment. Variations in the ORP together with an excess concentration of H_2_O_2_ can shift its role from a reducing agent to an oxidizing agent. Such condition may promote the re-oxidation of certain divalent, resulting in their precipitation and the formation of surface passivation layers, thereby hindering further metal dissolution (Miao et al., 2026). As 0.2 M H_2_O_2_ achieved metal recoveries comparable to those obtained with 1 and 2 M H_2_O_2_, higher concentrations were considered unnecessary. Similar observations were reported by Baniasadi et al. (2025), who investigated NMC leaching with biogenic acid containing different concentrations of H_2_O_2_ (0–5% v/v). In that study, 0.5% H_2_O_2_ was the optimal concentration, while increasing H_2_O_2_ concentrations led to a loss of Co and Ni at the beginning of the leaching (Baniasadi et al., 2025). Therefore, 0.2 M H_2_O_2_ was selected for all subsequent leaching experiments.

**FIGURE 2:**
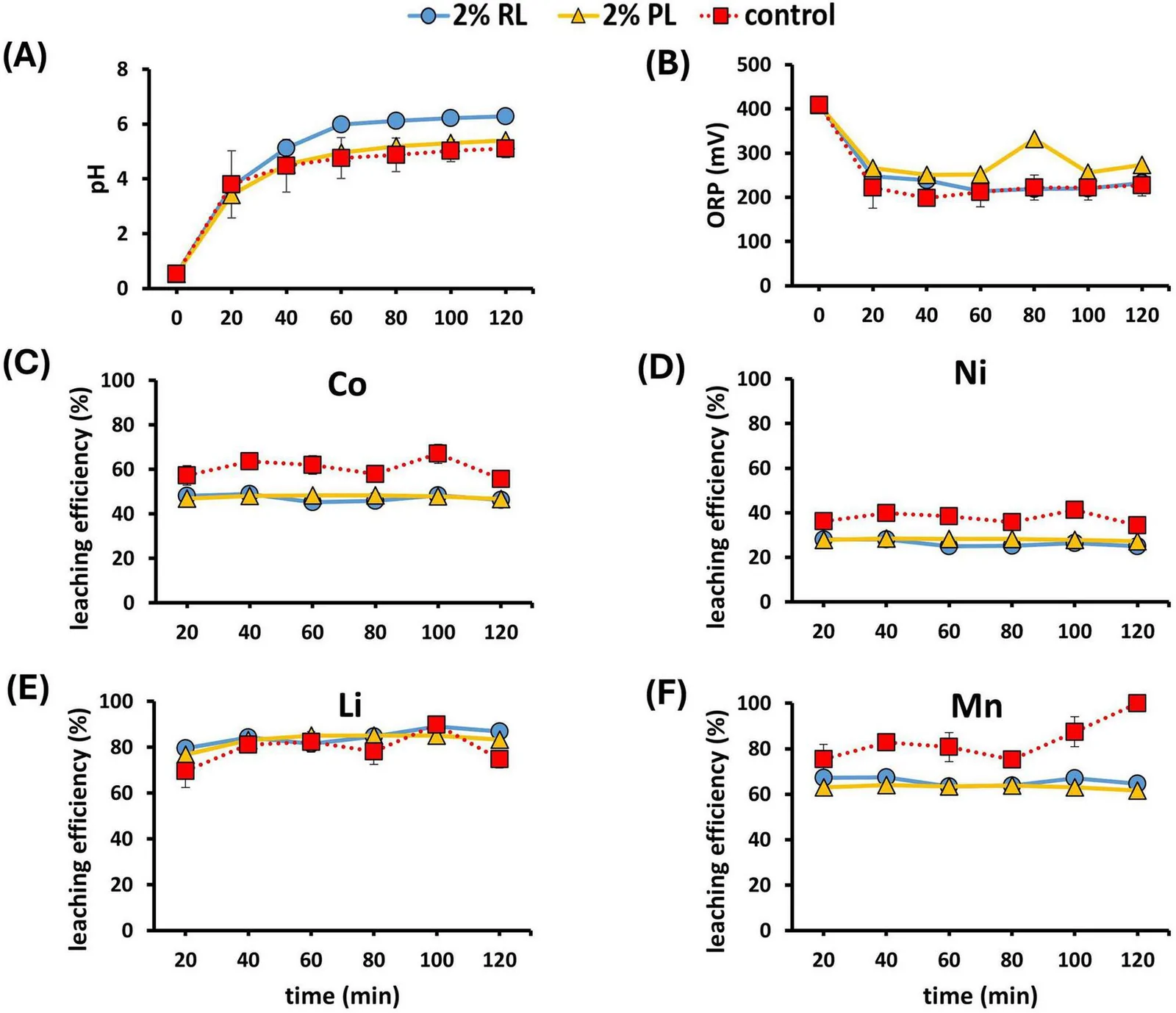
Changes in **(A)** pH **(B)** ORP, and leaching efficiencies of **(C)** Co, **(D)** Ni, **(E)** Li, and **(F)** Mn during indirect leaching of 10% (w/v) NMC using biogenic H_2_SO_4_ and 0.2 M H_2_O_2_, with additions of 2% (v/v) biosurfactants rhamnolipids (RL) and phospholipids (PL) after 40 min of leaching. H_2_SO_4_ –0.2 M H_2_O_2_ without biosurfactant is used as control. The error bars represent standard deviations (*n* = 2).

To investigate the effect of biosurfactants during BM leaching using biogenic acid and 0.2 M H_2_O_2_, 2% RL or 2% PL were added after 40 min of leaching, when the pH had exceeded 4 (Figure 2A), as biosurfactants generally exhibit greater stability at near-neutral pH (Abdel-Mawgoud et al., 2009). The addition of either 2% RL or 2% PL did not enhance metal solubilization despite the presence of 0.2 M H_2_O_2_, and instead, resulted in slightly lower recoveries of Co, Ni, and Mn compared with the control (H_2_SO_4_-H_2_O_2_ system) (Figures 2C–F). Furthermore, biosurfactant addition raised the solution pH, particularly in the PL-amended system, and partially changed the ORP profile shown in Figures 2A,B. These changes have likely affected the redox conditions required for efficient dissolution for transition metals from the NMC matrix. Biosurfactants possess a diverse array of functional groups, including hydroxyl, carboxyl, amino, carbonyl, and fatty acid moieties. These functional groups exhibit strong affinity to metal ions through multiple interaction mechanisms, including ion exchange, chelation, and electrostatic attraction (Yang et al., 2021). The changes in pH and ORP after the addition of PL and RL can be attributed to the ionizable functional groups present in the biosurfactant molecules and their interaction with dissolved metal species.

A recent study by Chakankar et al. (2026) investigated the use of rhamnolipids as floatation agents for metal recovery from a battery recycling process water and reported that the metal-binding affinity of rhamnolipid followed the order Mn^2+^>Ni^2+^>Co^2+^, whereas Li^+^ showed negligible interaction with the biosurfactant. These findings are consistent with the observations in the present study, where the addition of biosurfactants to the H_2_SO_4_–H_2_O_2_ system reduced the dissolution of Mn, Ni, and Co, while Li recovery remained largely unaffected (Figures 2C–F). The above findings suggest that interactions between dissolved metal ions and biosurfactants may lead to the formation of insoluble metal-biosurfactant aggregates, reducing thus the dissolution of Mn, Ni, and Co.

Rhamnolipids have been used for the removal of heavy metal contaminant such as Ni, Cu and Cd from manganese nodules (Lee and Kim, 2019). Biosurfactant-based emulsion liquid membrane proved to be an effective and cost-efficient treatment for the removal of Mn^2+^ from contaminated water, achieving more than 77% Mn removal in just 2 min (Ferreira et al., 2019). Tang et al. (2020) demonstrated that a combination of a biosurfactant and an electrokinetic technique enhances the removal of metals, including Ni, Mn, Cr, Pb, Zn and Cu, from sewage sludge. The biosurfactant surfactin has been successfully employed as a collector in ion floation, resulting in the recovery of 94.7 % Ni and 98.2% Co from synthetic wastewater solutions (Schlebusch et al., 2023). Therefore, biosurfactant have a potential for selective recovery of critical metals such as Co, Ni and Mn from leachate solution produced during biohydrometallurgical treatment of spent LIB. Further investigation should focus on evaluating broader range of biosurfactant and optimizing their concentrations to enhance metal recoveries and selectivity.

### 3.3 Scale up of H_2_SO_4_–H_2_O_2_ system

**FIGURE 3:**
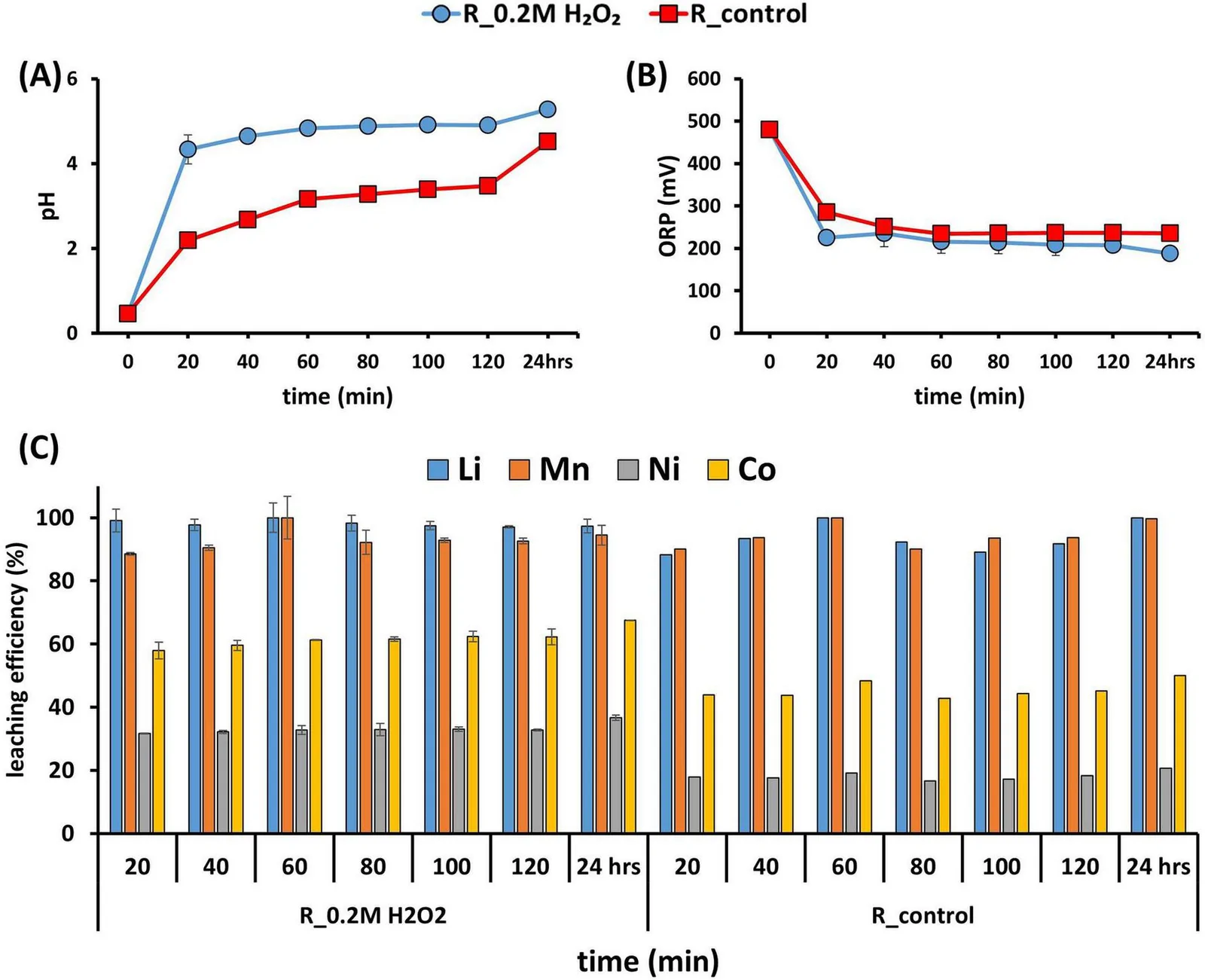
Changes in **(A)** pH and **(B)** ORP, and **(C)** leaching efficiencies of Li, Mn, Ni, and Co during upscaled indirect leaching of 10% (w/v) NMC using biogenic H_2_SO_4_ acid with 0.2 M H_2_O_2_ (R_0.2 M H_2_O_2_) without H_2_O_2_ (R_control). The error bars represent standard deviations (*n* = 2).

The indirect leaching of NMC using biogenic H_2_SO_4_ and 0.2 M H_2_O_2_ was scaled up to a 1 L reactor (Figure 3). Compared to the flask experiments (Figure 1), leaching efficiencies of Li and Mn increased significantly, likely due to enhanced mixing and improved solid-liquid contact under reactor conditions. The addition of H_2_O_2_ improved Co and Ni dissolution by ∼10% and ∼15%, respectively, compared to a H_2_O_2_-free control. In the H_2_O_2_-amended system, leaching efficiencies reached 61.2% for Co and 32.8% for Ni after 1 h, increasing further to 67.5% for Co and 36.6% for Ni after 24 h of leaching (Figure 3C). These results are consistent with (Meshram et al. (2016), who reported an increase in Co recovery from 66.2% to 79.2% upon addition of 5% H_2_O_2_ during NMC leaching with 1 M H_2_SO_4_. Miao et al. (2026) reported that dynamic dosing of H_2_O_2_, rather than a single step addition (as employed in the present study), maintains reducing condition throughout the leaching process, facilitating the complete reduction of Ni^3+^ and Co^3+^ to their divalent states. Therefore, adopting such dosing strategy could further improve the leaching efficiency, particularly for Co and Ni. For Li and Mn, complete recovery was achieved within 60 min in both H_2_O_2_-containing and H_2_O_2_-free systems, further supporting that proton attack alone is sufficient to promote their dissolution (Figure 3C). Similarly, during a scale-up (1 L) two-step bioleaching of NMC-based BM, in which the effect of magnetic field was investigated, complete recovery of Mn and Li was also achieved but with relatively low pulp density of 3% (w/v) and after 3 days of leaching time (Hong et al., 2025). Panda et al. (2024) successfully scale-up direct bioleaching of BM using an adapted consortium in a 10 L reactor, resulting in metal recoveries of up to 80% Li, 91% Ni, 75% Co and 75% Mn. Nevertheless, these recoveries were achieved using a relatively low pulp density of only 0.5 % (w/v), which may limit the industrial applicability of the process.

**FIGURE 4:**
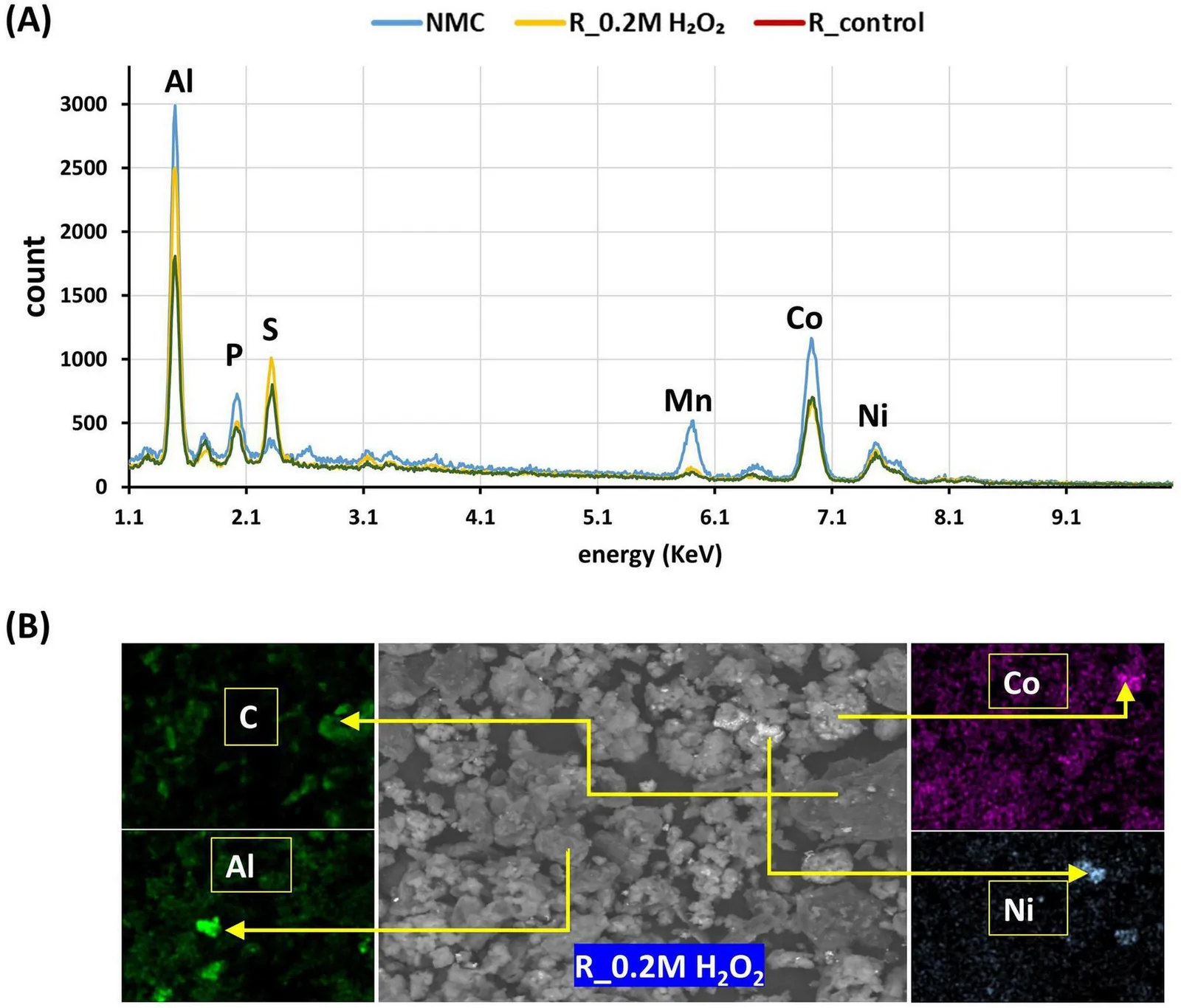
(A) SEM/EDS analysis of the original NMC-based black mass (NMC) and the solid residues collected after indirect leaching of 10% NMC using biogenic H_2_SO_4_ in a 1 L bioreactors, with (R_0.2 M H_2_O_2_) and without (control) 0.2 M H_2_O_2_; **(B)** SEM/EDS elemental mapping image (C, Al, Co, and N) of the solid residue collected from the reactor with 0.2 M H_2_O_2_.

SEM/EDS analysis of the solid residues collected after the reactor experiments confirmed complete removal of Mn from the solid phase, while residual Co, Ni, and Al were still detected in the post-leaching solids (Figure 4). In NMC structures, Mn is typically present as Mn^3+^ and Mn^4+^, and its reduction-dissolution to Mn^2+^ generally requires strongly acidic and reducing conditions. However, Meshram et al. (2015) reported that at pH > 3.2 and under low redox potential, Mn^4+^ can be indirectly solubilized via the formation of an intermediate Mn_2_O_3_ phase. The leaching of Mn in this study may therefore proceed through a similar pathway, i.e., through the formation of an intermediate phase, resulting in the high recovery of Mn in both reactor systems.

It has been reported that Cu and Al present in NMC can positively influence leaching behavior by acting as reducing agents that improve the overall metal dissolution (Joulié et al., 2017). The NMC used in this study contained relatively high concentrations of both Cu (3.5%) and Al (5.26%). However, Cu concentration in the leachates remained below the detection limit, while <40% of Al was dissolved in both reactors (results not shown). The limited Cu dissolution might be attributed to the absence of favorable redox cycling conditions (e.g., Fe^2+^/Fe^3+^ oxidation-reduction cycle), which are often associated with enhanced Cu mobilization. In contrast, Al behavior is highly pH-dependent, with precipitation expected to start occurring at pH 3–4, which may have limited its recovery under the conditions investigated. Therefore, optimization of the leaching conditions for both Cu and Al may further improve the recovery of Co and Ni (Porvali et al., 2020; Yan et al., 2023; Lalropuia et al., 2024).

Most previous studies on NMC leaching have been conducted at elevated temperatures (50–100 °C) and using concentrated acids (>1 M) (Baniasadi et al., 2025; Meshram et al., 2015; Mukai et al., 2024; Nadimi and Jalalian Karazmoudeh, 2020; Wang et al., 2016). In contrast, the present study operates at room temperature (22 °C) without external heating and uses dilute H_2_SO_4_ (0.6 M), thereby significantly reducing energy input and improving the potential economic viability of scale-up. The leaching agent, biogenic H_2_SO_4_, was produced from sulfur waste using chemolithotrophic bacteria that utilize CO_2_ as the sole carbon source, thereby enhancing process sustainability and potentially contributing to carbon neutral pathway. High recoveries of strategic metals, particularly Mn and Li, were achieved at a high pulp density of 10% (w/v) within 1 h of leaching. Overall, these findings demonstrate a scalable and sustainable bioleaching strategy for the efficient recovery of critical metals from NMC cathode waste. However, the incubation period for each cycle (14–21 days) is relatively long compared with conventional commercial acid production processes. Further optimization of the experimental condition is still necessary to reduce the incubation time and improve process efficiency. Välimets et al. (2026) reported that increasing the concentration of biogas derived sulfur substrate to 6% (w/v) resulted in an increase in acid concentration to as high as 2 M. The use of higher acid concentration employed in this study (0.6 M) could further enhance the kinetics of metal leaching from BM. Previous studies have demonstrated that bioleaching presents a cost-effective and environmentally friendly approach for metal recovery from different waste streams. Techno-economic analysis of LIB bioleaching using *Gluconobacter oxydans* demonstrated strong economic feasibility, achieving a positive net present value of $136 million and an 11% profit margin for a scale-up plant, while enabling efficient recovery of critical metals with a lower carbon footprint compared to conventional recycling processes (Khan et al., 2025). A sensitivity analysis conducted during techno-economic assessment of tungsten recovery from semiconductor wastewater via bioleaching highlighted pulp density and microbial cultivation time as key factor affecting the economic viability and scale-up feasibility (Lee et al., 2024). Furthermore, the current study does not include techno-economic analysis or life cycle analysis, both of which are essential for evaluating the economic feasibility and environmental sustainability of the proposed process and to enable comprehensive comparison with conventional LIB recycling technologies. Addressing these limitations in future studies could further improve the process efficiency, scalability, and practical applicability of biogenic acid-based LIB recycling.

## 4 Conclusion

Microbially produced H_2_SO_4_, using elemental sulfur recovered from a biogas plant, was successfully used for leaching black mass from NMC-based cathode material. The effect of varying H_2_O_2_ concentrations on metal solubilization was investigated, with 0.2 M identified as the optimum concentration for maximum metal extraction efficiency. Biosurfactants (rhamnolipids and phospholipids) were also evaluated in combination with H_2_O_2_, however, no improvement in metal leaching was observed. Finally, the optimized H_2_SO_4_-H_2_O_2_ was upscaled in a 1 L reactor, achieving 100% recovery of Mn and Li from 10% (w/v) NMC within 1 h. These findings underscore the high potential of biogenic acid for efficient recovery of critical metals from spent LIB. However, despite its high potential, further assessment of the economic feasibility and life cycle impacts is still required to validate this process as a cost effective and sustainable circular economy-based alternative for LIB recycling.

## Data Availability

The raw data supporting the conclusions of this article will be made available by the authors, without undue reservation.

## References

[B1] Abdel-Mawgoud A. M. Aboulwafa M. M. Hassouna N. A. (2009). Characterization of rhamnolipid produced by *Pseudomonas aeruginosa* isolate Bs20. *Appl. Biochem. Biotechnol*. 157 329–345. 10.1007/s12010-008-8285-1 18584127

[B2] Baniasadi M. Upvan K. Pourhossein F. Graves J. E. Latvyte E. Farnaud S. (2025). Towards a circular economy in lithium ion battery recycling by integrating microbial processes with electrowinning and precipitation for sustainable metal recovery. *J. Environ. Manage*. 395:127891. 10.1016/j.jenvman.2025.127891 41223505

[B3] Boxall N. J. Cheng K. Y. Bruckard W. Kaksonen A. H. (2018). Application of indirect non-contact bioleaching for extracting metals from waste lithium-ion batteries. *J. Hazard. Mater*. 360 504–511. 10.1016/j.jhazmat.2018.08.024 30144769

[B4] Chakankar M. Mongoljiibuu S. O. Matys S. Boelens P. Salces A. M. Kelly N. et al. (2026). Evaluation of biosurfactant-based ion flotation process for metal separation and selective recovery from battery recycling process waters. *J. Ind. Eng. Chem.* 10.1016/j.jiec.2026.06.016

[B5] Chernyshova I. Slabov V. Rao Kota H. (2023). Emerging application of biosurfactants in metal extraction Irina. *Curr. Opin. Colloid Interface Sci.* 68:101763. 10.1016/j.cocis.2023.101763

[B6] Diaz M. A. De Ranson I. U. Dorta B. Banat I. M. Blazquez M. L. Gonzalez F. et al. (2015). Metal removal from contaminated soils through bioleaching with oxidizing bacteria and rhamnolipid biosurfactants. *Soil Sediment Contam*. 24 16–29. 10.1080/15320383.2014.907239

[B7] Fallah N. Fitzpatrick C. (2023). Is shifting from Li-ion NMC to LFP in EVs beneficial for second-life storages in electricity markets? *J. Energy Storage* 68:107740. 10.1016/j.est.2023.107740

[B8] Ferreira L. C. Ferreira L. C. Cardoso V. L. Filho U. C. (2019). Mn(II) removal from water using emulsion liquid membrane composed of chelating agents and biosurfactant produced in loco. *J. Water Process Eng.* 29:100792. 10.1016/j.jwpe.2019.100792

[B9] Gu J. Nie Y. Li Z. Yang K. An L. Jiang H. et al. (2025). Efficient bioleaching of Li from waste lithium–iron phosphate batteries by acidophilic bacterial consortium: Enrichment condition, Li recovery, and brief carbon footprint analysis. *Chem. Eng. J.* 509:161450. 10.1016/j.cej.2025.161450

[B10] Hong J. H. Kim J. Han E. Yang S. M. Kim H. S. Kim J. et al. (2025). Magnetic field-assisted bioleaching of cathode materials from spent li-ion batteries using acidithiobacillus ferrooxidans. *Chemosphere* 376:144303. 10.1016/j.chemosphere.2025.144303 40081027

[B11] Joulié M. Billy E. Laucournet R. Meyer D. (2017). Current collectors as reducing agent to dissolve active materials of positive electrodes from li-ion battery wastes. *Hydrometallurgy* 169 426–432. 10.1016/j.hydromet.2017.02.010

[B12] Khan S. R. Khan M. M. Srivastava K. Jin H. Plante L. Lee J. J. et al. (2025). Sustainable recovery of critical metals from spent lithium-ion batteries through gluconic acid-based bioleaching: Techno-economic analysis, life cycle assessment and process optimization. *Chem. Eng. J.* 516:163714. 10.1016/j.cej.2025.163714

[B13] Kim J. Nwe H. H. Yoon C. S. (2024). Enhanced bioleaching of spent li-ion batteries using A. ferrooxidans by application of external magnetic field. *J. Environ. Manage*. 367:122012. 10.1016/j.jenvman.2024.122012 39094417

[B14] Kiskira K. Tsakanika L. A. Kritikos A. Papadopoulou K. Chatzitheodoridis E. Lyberatos G. et al. (2026). Microbial bioleaching of critical metals from spent lithium-ion batteries: A biohydrometallurgical approach. *Minerals* 16:277. 10.3390/min16030277

[B15] Kremser K. Maltschnig M. Schön H. Jandric A. Gajdosik M. Vaculovic T. et al. (2022). Optimized biogenic sulfuric acid production and application in the treatment of waste incineration residues. *Waste. Manag*. 144 182–190. 10.1016/j.wasman.2022.03.025 35378357

[B16] Kremser K. Schön H. Gerl P. Gómez M. ÁV. Espinosa D. R. Morandini A. et al. (2023). Pharmaceutical blister waste recycling using biogenic sulfuric acid: Effect of sulfur source and blister material on bioleaching efficiency. *Hydrometallurgy* 221:106124. 10.1016/j.hydromet.2023.106124

[B17] Lalropuia L. Kucera J. Rassy W. Y. Pakostova E. Schild D. Mandl M. et al. (2024). Metal recovery from spent lithium-ion batteries via two-step bioleaching using adapted chemolithotrophs from an acidic mine pit lake. *Front. Microbiol*. 15:1347072. 10.3389/fmicb.2024.1347072 38348186 PMC10861312

[B18] Lee A. Kim K. (2019). Removal of heavy metals using rhamnolipid biosurfactant on manganese nodules. *Water Air Soil Pollut.* 230:258. 10.1007/s11270-019-4319-2

[B19] Lee C. K. Rhee K.-I. (2003). Reductive leaching of cathodic active materials from lithium ion battery wastes. *Hydrometallurgy* 68 5–10. 10.1016/S0304-386X(02)00167-6

[B20] Lee C. Arby D. S. Kim C. Lim J. Kwon K. Chung E. (2025). Hydrometallurgical process of spent lithium-ion battery recycling Part. 1 Chemical leaching of valuable metals from cathode active materials: Review and case study. *Hydrometallurgy* 235:106494. 10.1016/j.hydromet.2025.106494

[B21] Lee Y. Choi H. Oh H. Chung S. Park J. Han J. (2024). Economic feasibility evaluation of bioleaching-based tungsten recycling of semiconductor industry waste. *ACS Sustain. Chem. Eng.* 12 10229–10238. 10.1021/acssuschemeng.4c02941

[B22] Liu A. Hu G. Wu Y. Guo F. (2024). Life cycle environmental impacts of pyrometallurgical and hydrometallurgical recovery processes for spent lithium-ion batteries: Present and future perspectives. *Clean Technol. Environ. Policy* 26 381–400. 10.1007/s10098-023-02640-x

[B23] Llamas-Orozco J. A. Meng F. Walker G. S. Abdul-Manan A. F. N. MacLean H. L. Posen I. D. et al. (2023). Estimating the environmental impacts of global lithium-ion battery supply chain: A temporal, geographical, and technological perspective. *PNAS Nexus*. 2:gad361. 10.1093/pnasnexus/pgad361 38034093 PMC10683946

[B24] Manthiram A. (2020). A reflection on lithium-ion battery cathode chemistry. *Nat. Commun*. 11:1550. 10.1038/s41467-020-15355-0 32214093 PMC7096394

[B25] Meshram P. Abhilash, Pandey B. D. Mankhand T. R. Deveci H. (2016). Comparision of different reductants in leaching of spent lithium ion batteries. *JOM* 68 2613–2623. 10.1007/s11837-016-2032-9

[B26] Meshram P. Pandey B. D. Mankhand T. R. (2015). Recovery of valuable metals from cathodic active material of spent lithium ion batteries: Leaching and kinetic aspects. *Waste. Manag*. 45 306–313. 10.1016/j.wasman.2015.05.027 26087645

[B27] Miao S. Fu M. Li X. Wu B. Wang Z. Li G. et al. (2026). Unraveling the synergistic mechanism of H2SO4/H_2_O_2_ system enabled by a dynamic dosing strategy for sustainable recycling of spent li-ion batteries. *Hydrometallurgy* 244 106811. 10.1016/j.hydromet.2026.106811

[B28] Mishra S. Lin Z. Pang S. Zhang Y. Bhatt P. Chen S. (2021). Biosurfactant is a powerful tool for the bioremediation of heavy metals from contaminated soils. *J. Hazard. Mater*. 418:126253. 10.1016/j.jhazmat.2021.126253 34119972

[B29] Mukai K. Takatani Y. Nonaka T. (2024). Mechanisms underlying the acid leaching process for LiNi0.6Co0.2Mn0.2O2 with and without H_2_O_2_. *Energy Adv.* 3 1099–1110. 10.1039/d4ya00049h

[B30] Mulligan C. N. Yong R. N. Gibbs B. F. (2001). Heavy metal removal from sediments by biosurfactants. *J. Hazard. Mater*. 85 111–125. 10.1016/s0304-3894(01)00224-2 11463506

[B31] Nadimi H. Jalalian Karazmoudeh N. (2020). Leaching of Co, Mn and Ni using H_2_O_2_ in sulfuric acid medium from mobile phone LIBs. *J. Inst. Eng. Ser. D* 101 111–116. 10.1007/s40033-020-00221-6

[B32] Ňancucheo I. Rowe O. F. Hedrich S. Johnson D. B. (2016). Solid and liquid media for isolating and cultivating acidophilic and acid-tolerant sulfate-reducing bacteria. *FEMS Microbiol. Lett*. 363:fnw083. 10.1093/femsle/fnw083 27036143

[B33] Naseri T. Mousavi S. M. (2024). Improvement of Li and Mn bioleaching from spent lithium-ion batteries, using step-wise addition of biogenic sulfuric acid by acidithiobacillus thiooxidans. *Heliyon* 10:e37447. 10.1016/j.heliyon.2024.e37447 39315164 PMC11417220

[B34] Pakostova E. Graves J. Latvyte E. Maddalena G. Horsfall L. (2024). A novel closed-loop biotechnology for recovery of cobalt from a lithium-ion battery active cathode material. *Microbiology* 170:001475. 10.1099/mic.0.001475 39016549 PMC11318048

[B35] Panda S. Dembele S. Mishra S. Akcil A. Agcasulu İ Hazrati E. et al. (2024). Small-scale and scale-up bioleaching of Li, Co, Ni and Mn from spent lithium-ion batteries. *J. Chem. Technol. Biotechnol.* 99 1069–1082. 10.1002/jctb.7609

[B36] Porvali A. Chernyaev A. Shukla S. Lundström M. (2020). Lithium ion battery active material dissolution kinetics in Fe(II)/Fe(III) catalyzed Cu-H2SO4 leaching system. *Sep. Purif. Technol.* 236:116305. 10.1016/j.seppur.2019.116305

[B37] Roy A. (2018). A review on the biosurfactants: Properties, types and its applications. *J. Fundamentals Renew. Energy Appl.* 08 1–5. 10.4172/2090-4541.1000248

[B38] Schlebusch I. Pott R. W. M. C. Tadie M. (2023). The ion flotation of copper, nickel, and cobalt using the biosurfactant surfactin. *Discov. Chem. Eng.* 3:7. 10.1007/s43938-023-00023-8

[B39] Tang J. He J. Tang H. Wang H. Sima W. Liang C. et al. (2020). Heavy metal removal effectiveness, flow direction and speciation variations in the sludge during the biosurfactant-enhanced electrokinetic remediation. *Sep. Purif. Technol.* 246:116918. 10.1016/j.seppur.2020.116918

[B40] Välimets S. Kalampaka A. Sumetzberger-Hasinger M. Lalropuia L. Ribitsch D. Guebitz G. M. (2026). Biogas-derived sulfur enhances microbial sulfuric acid production. *Waste Manag*. 224:115748. 10.1016/j.wasman.2026.115748 42468471

[B41] Vera M. Schippers A. Sand W. (2013). Progress in bioleaching: Fundamentals and mechanisms of bacterial metal sulfide oxidation–part A. *Appl. Microbiol. Biotechnol*. 97 7529–7541. 10.1007/s00253-013-4954-2 23720034

[B42] Vieceli N. Benjamasutin P. Promphan R. Hellström P. Paulsson M. Petranikova M. (2023). Recycling of lithium-ion batteries: Effect of hydrogen peroxide and a dosing method on the leaching of LCO, NMC oxides, and industrial black mass. *ACS Sustain. Chem. Eng.* 11 9662–9673. 10.1021/acssuschemeng.3c01238

[B43] Wang F. Sun R. Xu J. Chen Z. Kang M. (2016). Recovery of cobalt from spent lithium ion batteries using sulfuric acid leaching followed by solid-liquid separation and solvent extraction. *RSC Adv.* 6 85303–85311. 10.1039/c6ra16801a

[B44] Xin B. Zhang D. Zhang X. Xia Y. Wu F. Chen S. et al. (2009). Bioleaching mechanism of Co and Li from spent lithium-ion battery by the mixed culture of acidophilic sulfur-oxidizing and iron-oxidizing bacteria. *Bioresour. Technol*. 100 6163–6169. 10.1016/j.biortech.2009.06.086 19656671

[B45] Yan K. Chen Q. Zhang Z. Nie H. Wang R. Xu Z. (2023). A closed-loop process for high-value regeneration of spent LiFePO4 cathodes after selective aluminium precipitation. *Green Chem.* 25 9156–9166. 10.1039/d3gc03144f

[B46] Yang Z. Wang D. Wang G. Zhang S. Cheng Z. Xian J. et al. (2021). Removal of Pb, Zn, Ni and Cr from industrial sludge by biodegradable washing agents: Caboxyethylthiosuccinic acid and itaconic-acrylic acid. *J. Environ. Chem. Eng.* 9:105846. 10.1016/j.jece.2021.105846

[B47] Zanoletti A. Carena E. Ferrara C. Bontempi E. (2024). A review of lithium-ion battery recycling: Technologies, sustainability, and open issues. *Batteries* 10:38. 10.3390/batteries10010038

